# APC is epigenetically down regulated in advance cases of gallbladder cancer

**DOI:** 10.1186/1755-8166-7-S1-P21

**Published:** 2014-01-21

**Authors:** Dinesh Tekcham Singh, Satish Poojary, Shushruta Bhunia, Mustafa Ahmed Barbhuiya, Manisha Kakkar, Vishwajit Jalaj, Sanjeev Gupta, Braj Raj Shrivastav, Pramod Kumar Tiwari

**Affiliations:** 1 Centre for Genomics, Molecular and Human Genetics, School of Studies in Zoology, Jiwaji University, Gwalior-474011, India; 2 Cancer Hospital and Research Institute, Gwalior-474007, India; 3 Gajra Raja Medical College, Gwalior-474007, India

## Background

The mortality rate of gallbladder cancer (GBC) is considerably high in India and world over. The Adenomatous Polyposis Coli (APC) gene is widely reported for its role in cancer. APC is known to have two promoter regions, 1A and 1B, that show differential role in various cancers. However, role of these promoters and their exons in the molecular pathogenesis of GBC is obscure. Our aim was to study the epigenetic control of APC promoters in GBC and to evaluate their utility as prognostic/diagnostic biomarker of GBC.

## Methods

We carried out methylation specific PCR of the modified genomic DNA from GBC and GSD tissues, followed by semi-quantitative and quantitative PCRs (qPCR). We compared the transcript levels of the two exons of APC in GBC and GSD as compared to their adjacent control tissues. We also performed Immunohistochemistry (IHC) on tissue micro array (TMA) of GBC and GSD. The scoring of different tissue cores was done by the expert pathologist and student’s t-test was performed to check the significance.

## Results

The APC 1A promoter was found significantly methylated in both GBC (96%; p=0.0155) and GSD (80%; p=0.015), but 1B was not. The down-regulation of APC exon 1 was observed in both GBC and GSD, whereas exon 2 appeared normally expressed. Immunohistochemistry of APC protein on Tissue Microarray (TMA) revealed down regulation with negative score of 34.48% in GBC (p=0.057), 1+ in 24.14% GBC (p=0.005), 2+ in 25.85% in early stage or GSD (p=0.091).

## Conclusion

We infer that APC is epigenetically down regulated in advance cases of GBC and might be a key step in the tumorigenesis of gallbladder. In future, APC may be considered for diagnostic, prognostic and therapeutic purposes in GBC.

